# Identification of hub biomarkers of myocardial infarction by single-cell sequencing, bioinformatics, and machine learning

**DOI:** 10.3389/fcvm.2022.939972

**Published:** 2022-07-25

**Authors:** Qunhui Zhang, Yang Guo, Benyin Zhang, Hairui Liu, Yanfeng Peng, Di Wang, Dejun Zhang

**Affiliations:** ^1^Research Center for High Altitude Medicine, Key Laboratory of High-Altitude Medicine (Ministry of Education), Key Laboratory of Application and Foundation for High Altitude Medicine Research in Qinghai Province (Qinghai-Utah Joint Research Key Lab for High Altitude Medicine), Qinghai University, Xining, China; ^2^College of Eco-Environmental Engineering, Qinghai University, Xining, China

**Keywords:** myocardial infarction, gene, single-cell sequencing, machine learning, immune infiltration, drug prediction, molecular docking

## Abstract

**Background:**

Myocardial infarction (MI) is one of the first cardiovascular diseases endangering human health. Inflammatory response plays a significant role in the pathophysiological process of MI. Messenger RNA (mRNA) has been proven to play a key role in cardiovascular diseases. Single-cell sequencing (SCS) technology is a new technology for high-throughput sequencing analysis of genome, transcriptome, and epigenome at the single-cell level, and it also plays an important role in the diagnosis and treatment of cardiovascular diseases. Machine learning algorithms have a wide scope of utilization in biomedicine and have demonstrated superior efficiency in clinical trials. However, few studies integrate these three methods to investigate the role of mRNA in MI. The aim of this study was to screen the expression of mRNA, investigate the function of mRNA, and provide an underlying scientific basis for the diagnosis of MI.

**Methods:**

In total, four RNA microarray datasets of MI, namely, GSE66360, GSE97320, GSE60993, and GSE48060, were downloaded from the Gene Expression Omnibus database. The function analysis was carried out by Gene Ontology (GO), Kyoto Encyclopedia of Genes and Genomes (KEGG), and Disease Ontology (DO) enrichment analysis. At the same time, inflammation-related genes (IRGs) were acquired from the GeneCards database. Then, 52 co-DEGs were acquired from differentially expressed genes (DEGs) in differential analysis, IRGs, and genes from SCS, and they were used to construct a protein-protein interaction (PPI) network. Two machine learning algorithms, namely, (1) least absolute shrinkage and selection operator and (2) support vector machine recursive feature elimination, were used to filter the co-DEGs. Gene set enrichment analysis (GSEA) was performed to screen the hub-modulating signaling pathways associated with the hub genes. The results were validated in GSE97320, GSE60993, and GSE48060 datasets. The CIBERSORT algorithm was used to analyze 22 infiltrating immune cells in the MI and healthy control (CON) groups and to analyze the correlation between these immune cells. The Pymol software was used for molecular docking of hub DEGs and for potential treatment of MI drugs acquired from the COREMINE.

**Results:**

A total of 126 DEGs were in the MI and CON groups. After screening two machine learning algorithms and key co-DEGs from a PPI network, two hub DEGs (i.e., IL1B and TLR2) were obtained. The diagnostic efficiency of IL1B, TLR2, and IL1B + TLR2 showed good discrimination in the four cohorts. GSEA showed that KEGG enriched by DEGs were mainly related to inflammation-mediated signaling pathways, and GO biological processes enriched by DEGs were linked to biological effects of various inflammatory cells. Immune analysis indicated that IL1B and TLR2 were correlated with various immune cells. Dan shen, san qi, feng mi, yuan can e, can sha, san qi ye, san qi hua, and cha shu gen were identified as the potential traditional Chinese medicine (TCM) for the treatment of MI. 7-hydroxyflavone (HF) had stable combinations with IL1B and TLR2, respectively.

**Conclusion:**

This study identified two hub DEGs (IL1B and TLR2) and illustrated its potential role in the diagnosis of MI to enhance our knowledge of the underlying molecular mechanism. Infiltrating immune cells played an important role in MI. TCM, especially HF, was a potential drug for the treatment of MI.

## Introduction

Myocardial infarction (MI), a serious cardiovascular disease caused by the rupture of unstable plaque, endangers human health worldwide. The mortality rate and the disease burden of MI are still in rapid growth, especially in China (1, 2). The early diagnosis of MI and the initiation of interventional treatment can decrease apoptotic cardiomyocytes, enhance prognosis, and reduce mortality (3). It is well known that the gold standard for diagnosing MI is evaluating the levels of markers of myocardial injury. However, these biomarkers do not quickly reflect the status of patients with MI due to their lack of sensitivity and specificity, leading patients to miss the best time for treatment (4). Consequently, it is essential to identify the underlying novel biomarkers in the diagnosis and treatment of MI to reduce premature mortality and improve prognosis.

A growing amount of evidence suggests that hereditary factors play an essential role in the development of MI progression. Genome-wide association studies (GWAS) have detected many susceptibility loci of MI (5). However, the outcomes of GWAS have failed to disclose the relevant risk of MI. Only a small fraction of these alterations can be used to explain the pathogenesis and progression of MI. Single-cell sequencing (SCS) technology is a new technology for the high-throughput sequencing analysis of genome, transcriptome, and epigenome at the single-cell level, and it also plays an important role in the diagnosis and treatment of cardiovascular diseases (6, 7). Machine learning plays an important role in screening for hub genes in gene chips.

Inflammation is associated with the occurrence of many cardiovascular diseases, especially MI (8). The inflammatory response cascade is essential in cardiac repair, remodeling, and fibrosis after MI, and non-selective suppression of inflammation after MI may detrimentally favor scarring, promote rupture, and exacerbate adverse remodeling (9). In addition, encouraging results from clinical trials of canakinumab have raised hopes for a therapy targeting inflammation in patients with MI (10, 11). What gene is involved in the regulation of MI through which inflammatory response is induced, and the specific mechanism of regulation are still unclear and need to be further explored.

Consequently, in this study, RNA microarray datasets were downloaded from the Gene Expression Omnibus (GEO) database to identify differentially expressed genes (DEGs). Subsequently, inflammation-related genes (IRGs) were obtained from the GeneCards online platform. In addition, SCS genes were acquired from the Human Cell Landscape (HCL) online platform. Co-genes were used to construct a protein-protein interaction (PPI) network and perform GSEA. Furthermore, two machine learning algorithms were used to identify the hub genes. At the same time, the validation of hub genes was carried out in three independent MI cohorts. In addition, immuno-infiltration analysis was carried out to investigate the relationship between the immune cell and hub genes. Finally, COREMINE online platform was used to obtain the predictive drugs, and molecular docking was used to verify.

## Materials and methods

### Myocardial infarction studies collection

**FIGURE 1:**
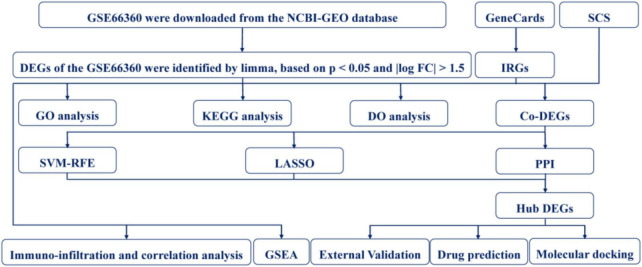
The flowchart of this study.

The design of this study is shown in Figure 1. Four RNA microarray datasets, namely, GSE66360, GSE97320, GSE60993, and GSE48060, were acquired from the National Center for Biotechnology Information’s Gene Expression Omnibus (GEO) database. The data of GSE66360, GSE97320, and GSE48060 were collected from the Affymetrix Human Genome U133 Plus 2.0 Array (HG-U133_Plus_2). The data of GSE60993 was collected from the Illumina Human WG-6 v3.0 Expression Beadchip. Finally, we selected 50 MI samples and 49 healthy control (CON) samples in the GSE66360 for subsequent analysis.

### Differentially expressed genes collection

The DEGs were acquired using the R package “limma” (12). Samples with *P* < 0.05 and | log FC| > 1.5 were regarded as the threshold for DEGs. PCA was used to evaluate the efficiency of DEGs (13).

### Single-cell data analysis

The human cell landscape online platform^1^ (14) was used to investigate the expression landscape of target genes in human normal adult heart tissue from published results of SCS (Supplementary Table 1).

### Inflammation-related genes collection

The IRGs (Supplementary Table 2) were acquired from the GeneCards database^2^ (15).

### Enrichment analysis

Subsequently, Gene Ontology (GO), Kyoto Encyclopedia of Genes and Genomes (KEGG), and Disease Ontology (DO) enrichment analyses were performed using the R package “clusterProfiler” (16) or Metascape^3^ online platform.

### Protein-protein interaction network construction

The intersection genes of DEGs, SCS, and IRGs were considered co-DEGs expressed in patients with MI. These results were used for a subsequent study. A protein-protein interaction network of co-DEGs was constructed using the STRING online platform^4^ (17). According to the degree of the PPI network, the significance of intersection genes was acquired (18). The correlation among co-DEGs was evaluated using the Pearson’s correlation analysis, which was calculated using Rstudio (Version 1.4.1717) (19). This result was visualized using Cytoscape (Version 3.6.1) (20).

### Diagnostic hub differentially expressed genes screening

To find significant prognostic variables, two different algorithms were used to predict the status of the disease. The least absolute shrinkage and selection operator (LASSO) is a kind of regression analysis algorithm used to enhance the prediction accuracy (21). The LASSO regression analysis was conducted using the R package “glmnet” to identify the crucial genes connected with the discrimination of MI and CON (22). Support vector machine (SVM) is also a technique of supervised machine learning that is widely used for classification and regression (23). Recursive feature elimination (RFE) is an algorithm used to choose the optimal genes from samples (24). So, SVM-RFE was applied to select the superior features. The candidate diagnostic overlapping genes were acquired from two algorithms and the above PPI network.

### Gene set enrichment analysis

The GSEA function was performed to identify the biological functions of the DEGs using the GO gene set (c5.go.v7.4.symbols.gmt) and KEGG gene set (c2.cp.kegg.v7.4.symbols.gmt). The threshold was set as *P* < 0.05. The enrichment diagrams were plotted using the R packages “clusterProfiler,” “enrichplot,” and “org.Hs.eg.db.”

### Diagnostic value of hub differentially expressed genes in myocardial infarction

To detect the predictive value of the ascertained biomarkers, we produced a receiver operator characteristic (ROC) curve using the expression data of mRNA from 50 MI and 49 CON samples. The area under the curve (AUC) from ROC was used to assess the effectiveness in the discrimination of MI and CON groups and further validated in the GSE97320, GSE60993, and GSE48060 datasets.

### Immuno-infiltration analysis

To better understand the proportions of infiltrating immune cells in the MI and CON groups, the CIBERSORT^5^ algorithm was used to calculate the infiltrating immune cells (25). A comparison of the infiltrated immune cell fractions in the MI and CON groups was performed using the Wilcoxon test with a significant *P* < 0.05.

### Immune correlation analysis

The correlation of infiltrating immune cells was assessed using Pearson’s correlation coefficient test (26). The relationship between hub DEGs and vital infiltrated immune cells was identified by Pearson’s correlation analysis of the R package “corrplot.” *P* < 0.05 was regarded as a statistically significant difference.

### Drug prediction

The hub DEGs were input into the COREMINE^6^ online platform to acquire the underlying treatment of MI. The underlying drugs were acquired. The threshold was set as *P* < 0.05.

### Molecular docking

Protein target receptors for macromolecules were acquired from the RCSB^7^ online platform. AutoDock Vina (1.5.6) was used to obtain a binding model prediction. PyMOL (1.7.x) was used to visualize the results.

### Statistical analysis

All statistical analysis was performed using Rstudio (Version 1.4.1717). *P* < 0.05 and |log FC| > 1.5 were used as the filtering criteria for DEGs. Pearson’s correlation analysis was carried out to find the gene co-expression. *P* < 0.05 was regarded as statistical significance.

## Results

### Identification of differentially expressed genes in patients with myocardial infarction

**FIGURE 2:**
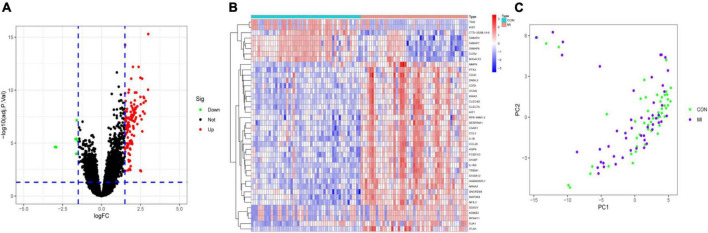
Identification of DEGs. **(A)** The volcano plot presented the DEGs between MI and CON groups. The upregulated genes are labeled in red, and the downregulated genes are labeled in green. **(B)** The heatmap visualized the alterations in the expression of DEGs. **(C)** PCA of DEGs showed good differentiation power.

Differential analysis was performed to identify DEGs in patients with MI and CON. The threshold was set as *P* < 0.05 and |log FC| > 1.5. A total of 126 DEGs were identified. Among them, 118 were upregulated and 8 were downregulated (Figures 2A,B). PCA showed that these DEGs could allow differentiation between MI and CON groups (Figure 2C).

### Enrichment analyses

**FIGURE 3:**
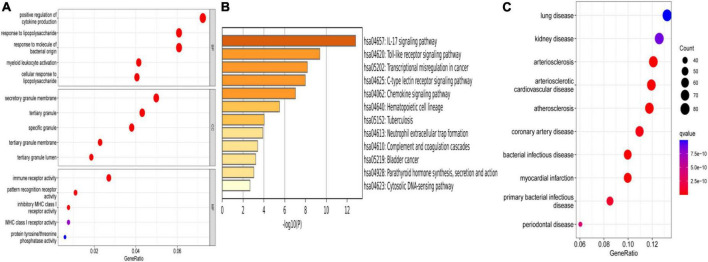
Enrichment analysis. **(A)** GO enrichment analysis of DEGs. **(B)** KEGG enrichment analysis of DEGs. **(C)** DO enrichment analysis of DEGs.

To explore the function of these DEGs, GO, KEGG, and DO enrichment analyses were conducted using the R package “clusterProfiler.” GO analysis showed that the GO biological processes included positive regulation of cytokine production, response to lipopolysaccharide, and response to molecule of bacterial origin. GO cellular component contained secretory granule membrane, tertiary granule, and specific granule. GO molecular function included immune receptor activity, pattern recognition receptor activity, and inhibitory MHC class I receptor activity (Figure 3A). KEGG analysis showed that these DEGs took part in the IL-17 signaling pathway, toll-like receptor signaling pathway, and chemokine signaling pathway (Figure 3B). According to DO enrichment, these DEGs were involved in arteriosclerotic cardiovascular disease, coronary heart disease, and MI (Figure 3C). These results demonstrated a tight correlation between DEGs and MI and that DEGs primarily mediated inflammatory responses.

### Identification of co-differentially expressed genes and protein-protein interaction network construction

**FIGURE 4:**
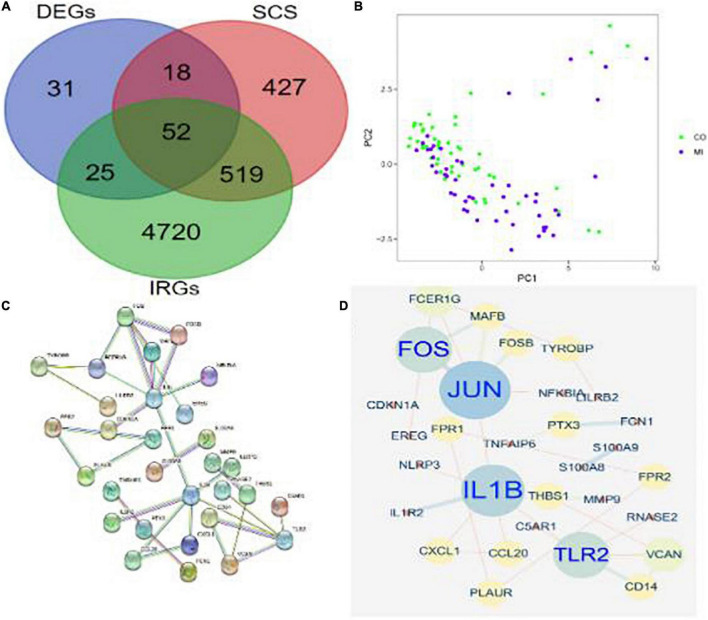
Identification of co-DEGs in patients with MI. **(A)** A Venn diagram showed the intersection of obtained DEGs, SCS, and IRGs, where 52 co-DEGs were identified. **(B)** PCA of co-DEGs showed good differentiation power. **(C)** A PPI network diagram of 52 co-DEGs was obtained from the STRING online platform. **(D)** Another PPI network diagram of 52 co-DEGs was constructed using Cytoscape 3.6.1.

The DEGs in differential analysis, DEGs in SCS, and acquired IRGs were integrated. Subsequently, 52 co-DEGs were obtained in patients with MI (Figure 4A). In addition, PCA showed that these genes could differentiate between MI and CON groups (Figure 4B). A PPI network diagram of 52 co-DEGs was constructed using the STRING online platform with combined scores of > 0.9 points (Figure 4C and Supplementary Table 3). The former result was put into Cytoscape to construct another PPI diagram, consisting of 30 nodes and 32 edges (Figure 4D).

### Functional analysis of differentially expressed genes by gene set enrichment analysis

**FIGURE 5:**
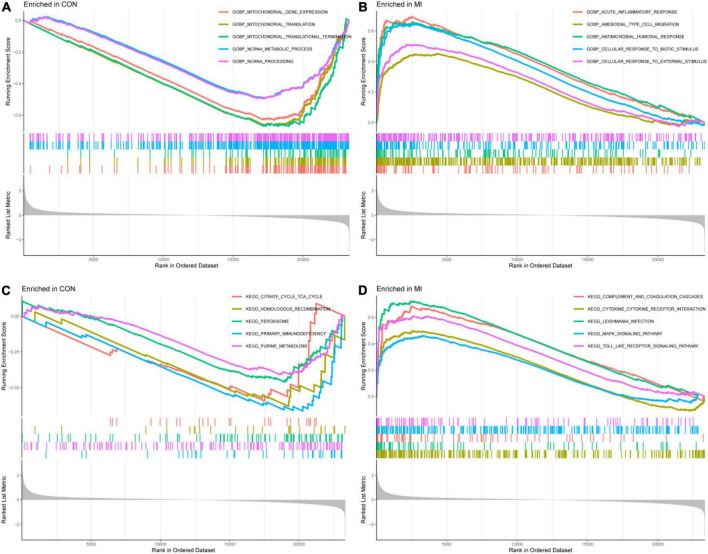
The results of GSEA. **(A,B)** The GSEA results from the GSE66360 dataset in GO analysis. **(C,D)** The GSEA results from the GSE66360 dataset in KEGG analysis.

The results of GSEA of GOBP were enriched in the acute inflammatory response, ameboidal type cell migration, and cellular response to external stimulus in the MI group and mitochondrial gene expression, mitochondrial translation, and NC RNA metabolic process in the CON group (*P* < 0.05, Figures 5A,B). The results of GSEA of KEGG were enriched in complement and coagulation cascades, MAPK signaling pathway, toll-like receptor signaling pathway, non-homologous end joining, proteasome, and protein export in the MI and CON groups (*P* < 0.05, Figures 5C,D).

### Identification and validation of hub differentially expressed genes in patients with myocardial infarction

**FIGURE 6:**
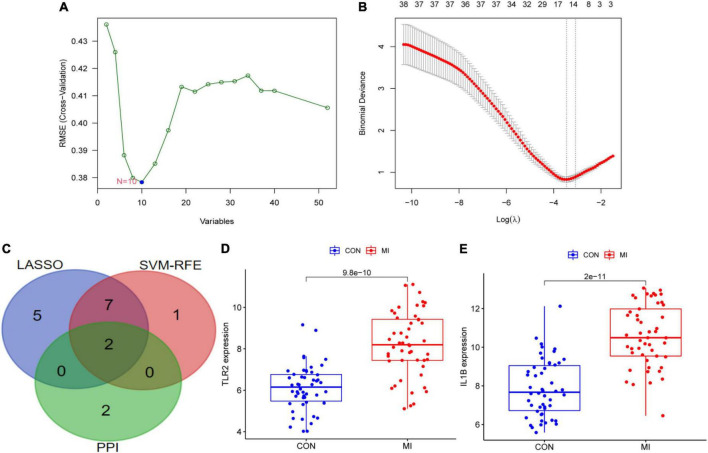
The screening of hub DEGs using machine learning and PPI. **(A)** 10 DEGs were obtained using the SVM-RFE algorithm. **(B)** 14 DEGs were obtained using the LASSO regression algorithm. **(C)** A Venn diagram presented the hub DEGs. **(D)** The expression levels of TLR2 in GSE66360. **(E)** The expression levels of IL1B in GSE66360.

Two different algorithms (SVM-RFE and LASSO) were conducted to screen the underlying feature biomarkers, yielding 10 and 14 genes, respectively (Figures 6A,B and Supplementary Table 4). A Venn diagram showed the intersection of obtained two hub DEGs (i.e., IL1B and TLR2) of SVM-RFE, LASSO, and PPI network (Figure 6C). The expression levels of IL1B and TLR2 in the MI group were remarkably higher than those in the CON group (Figures 6D,E).

### Diagnostic efficiency of IL1B, TLR2 and IL1B+TLR2 in patients with myocardial infarction

**FIGURE 7:**
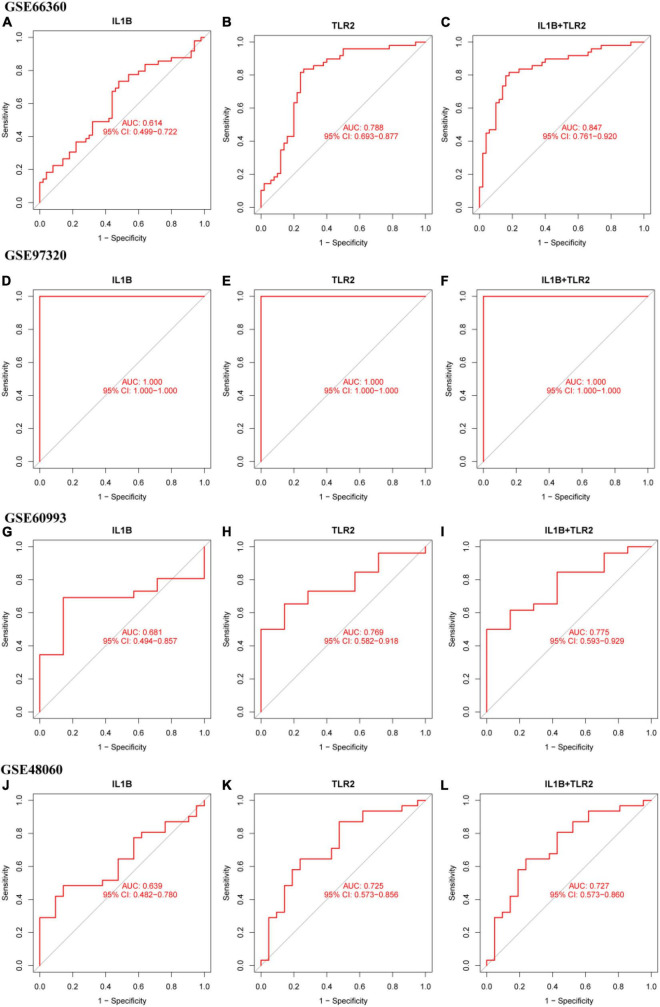
The ROC curve of the diagnostic efficiency of IL1B, TLR2, and IL1B + TLR2. **(A)** ROC curve of IL1B in the GSE66360. **(B)** ROC curve of TLR2 in the GSE66360. **(C)** ROC curve of IL1B + TLR2 in the GSE66360. **(D)** ROC curve of IL1B in the GSE97320. **(E)** ROC curve of TLR2 in the GSE97320. **(F)** ROC curve of IL1B + TLR2 in the GSE97320. **(G)** ROC curve of IL1B in the GSE60993. **(H)** ROC curve of TLR2 in the GSE60993. **(I)** ROC curve of IL1B + TLR2 in the GSE60993. **(J)** ROC curve of IL1B in the GSE48060. **(K)** ROC curve of TLR2 in the GSE48060. **(L)** ROC curve of IL1B + TLR2 in the GSE48060.

The diagnostic efficiency of the IL1B, TLR2, and IL1B + TLR2 in the discrimination of MI and CON groups indicated a good diagnostic value, with the AUC of 0.614 (95%CI: 0.499–0.722), 0.788 (95%CI: 0.693–0.877), and 0.847 (95%CI: 0.781-0.920) in the GSE66360 (Figures 7A–C). In addition, a good discrimination ability was testified in the GSE97320, GSE60993, and GSE48060 datasets. The diagnostic efficiency of the IL1B, TLR2, and IL1B + TLR2 in the discrimination of MI and CON groups indicated a good diagnostic value, with the AUC of 1.000 (95%CI: 1.000–1.000), 1.000 (95%CI: 1.000–1.000), and 1.000 (95%CI: 1.000–1.000) in the GSE97320 (Figures 7D–F). The diagnostic efficiency of the IL1B, TLR2, and IL1B + TLR2 in the discrimination of MI and CON groups indicated a good diagnostic value, with the AUC of 0.681 (95%CI: 0.494–0.857), 0.769 (95%CI: 0.582–0.918), and 0.775 (95%CI: 0.593–0.929) in the GSE60993 (Figures 7G–I). The diagnostic efficiency of the IL1B, TLR2, and IL1B + TLR2 in the discrimination of MI and CON groups indicated a good diagnostic value, with the AUC of 0.638 (95%CI: 0.482–0.780), 0.725 (95%CI: 0.473–0.856), and 0.727 (95%CI: 0.573–0.860) in the GSE48060 (Figures 7J–L).

### Immune infiltration analysis

**FIGURE 8:**
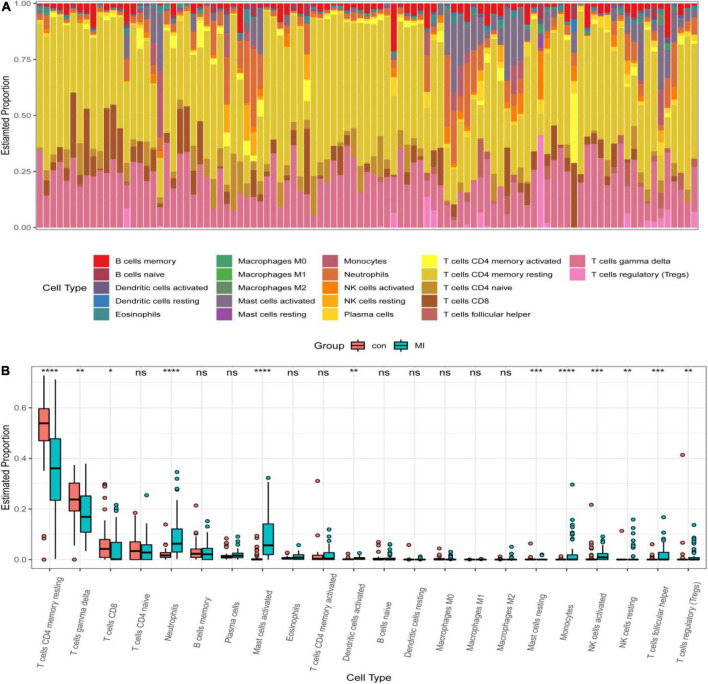
Distribution of infiltrating immune cells. **(A)** A heatmap of the infiltrating immune cells. **(B)** Comparison of 22 immune cells between MI and CON groups. Red and green colors denote CON and MI groups, respectively.

IL1B and TLR2 played an important role in the process of MI. So, it is necessary to investigate the relationship between hub DEGs and immune cells. CIBERSORT algorithm was used to explore the immune cell infiltration. Reasonably, the results indicated that there was a marked difference in the proportions of immune cells (Figure 8A). Then, we compared the difference in immune cell infiltration between the MI and CON groups. As depicted in the picture, compared with the CON group, the proportions of “monocytes,” “activated dendritic cells,” “activated mast cells,” “activated NK cells,” “resting NK cells,” “T cells follicular helper,” “regulatory T cells (Tregs),” and “neutrophils” (*P* < 0.001) were highly expressed in the MI group (Figure 8B). However, the proportions of “CD 4 resting memory T cells,” “gamma delta T cells,” “CD8 T cells,” and “resting mast cells” (*P* < 0.001) were significantly lower than that in the CON group (Figure 8B).

### Immune correlation analysis

**FIGURE 9:**
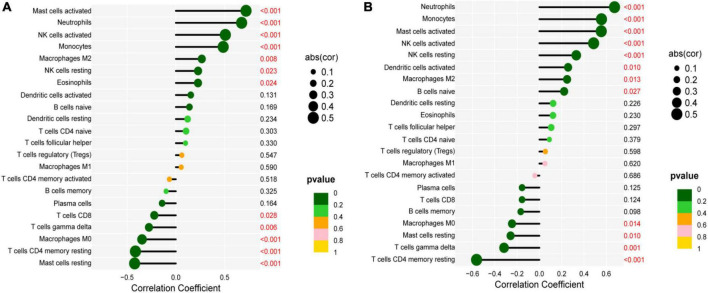
Correlation between IL1B, TLR2, and infiltrating immune cells. **(A)** Correlation between IL1B and infiltrating immune cells. **(B)** Correlation between TLR2 and infiltrating immune cells.

The correlation between IL1B, TLR2, and infiltrating immune cells was assessed. IL1B was positively correlated with activated mast cells, neutrophils, activated NK cells, monocytes, M2 macrophages, resting NK cells, and eosinophils (*r* > 0, *P* < 0.05), but significantly negatively correlated with resting mast cells, CD 4 resting memory T cells, M0 macrophages, gamma delta T cells, and CD8 T cells (*r* < 0, *P* < 0.05, Figure 9A). TLR2 was positively correlated with neutrophils, monocytes, activated mast cells, activated NK cells, resting NK cells, activated dendritic cells, M2 macrophages, and native B cells (*r* > 0, *P* < 0.05), but significantly negatively correlated with CD 4 resting memory T cells, gamma delta T cells, resting mast cells, and M0 macrophages (*r* < 0, *P* < 0.05, Figure 9B).

### Drug prediction

The IL1B, TLR2, and MI were input into the COREMINE online platform. The threshold was set as *P* < 0.05. The results demonstrated that dan shen, san qi, feng mi, yuan can e, can sha, san qi ye, san qi hua, and cha shu gen were identified as the potential traditional Chinese medicine (TCM) for the treatment of MI.

### Molecular docking

In our previous study, *Oxytropis falcata* Bunge (*O. falcata*), a Tibetan medicine/TCM, has played a role in anti-myocardial ischemia-reperfusion injury (MI/RI), and 7-hydroxyflavone (HF) was likely to be proven as a pharmacodynamic substance against MI/RI. Thus, we provided an experimental basis for its early clinical application by simulating molecular docking. The results showed that HF and IL-1B had stable combinations, and the binding affinity was −6.7 kcal/mol. Furthermore, HF and TLR2 had stable combinations, and the binding affinity was −7.4 kcal/mol.

## Discussion

Myocardial infarction, a common epidemic coronary heart disease in the world, can lead to sudden major adverse cardiovascular events (MACE), causing high mortality and disability (4). Thus, patients with MI show poor clinical prognosis. Due to the lack of a valid early diagnosis, MI patients often lose effective treatment, contributing to a poor prognosis. Early diagnosis can effectively improve the prognosis and reduce mortality in MI patients (2). The boom in microarray technology offers opportunities for the treatment of MI (27). More importantly, machine learning algorithms have a wide scope of utilization in biomedicine and have demonstrated superior efficiency in clinical trials (28). Furthermore, infiltrating immune cells have been proved to play a significant role in MI (29). However, few studies have paid attention to integration with these methods to investigate the association between biomarkers and infiltrating immune cells in patients with MI. Consequently, two machine learning algorithms were used to screen candidate diagnostic biomarkers for MI and explore the role of infiltrating immune cells in MI patients.

In this study, we systematically filtered two hub DEGs specifically expressed in MI patients using two machine learning algorithms and a PPI network to build a superior MI diagnostic model. Furthermore, the diagnostic efficiency of the diagnostic model was assessed in three independent cohorts. The outcomes of enrichment analysis showed that diseases enriched by DEGs were primarily associated with MI and arteriosclerotic cardiovascular disease, KEGG enriched by DEGs were mainly related to inflammation-mediated signaling pathways, and GO biological processes enriched by DEGs were linked to biological effects of various inflammatory cells. These results are in agreement with the former study that leukocyte-mediated inflammatory responses are involved in the pathophysiological process of MI (30). It is well known that MI is caused by unstable and vulnerable plaques and is regarded as a long-term chronic inflammatory disorder (31). In the acute phase of MI, a massive inflammatory response is triggered by the sudden cessation of blood flow, leading to MI. When MI occurs, abnormal accumulation of inflammatory cells and cytokines, platelet aggregation, rupture of vulnerable plaques, and apoptosis of cardiac myocytes have been implicated in the mechanism (32). TNF has been proved to participate in inflammatory response by the combination with its specific receptors in the development of MI. This evidence is consistent with our findings, supporting that the results of this study are precise, as well as indicating that immune response plays a crucial role in MI. The inflammatory immune response plays a crucial role in MI. It is necessary to control all kinds of immune cells accurately to accomplish a much more safe and more effective treatment (33). Consequently, the acquisition of novel biomarkers of MI same as the magnitude of infiltrating immune cells and IRGs by bioinformatics and SCS will make contributions to its effective treatment.

Inflammation is associated with the occurrence of MI. Thus, based on the three machine learning algorithms, one diagnostic hub IRG was identified. Interleukin-1B (IL1B), a core proinflammatory cytokine, is a member of the interleukin 1 (IL-1) cytokine family and is involved in a variety of inflammatory responses and cellular activities, including cell proliferation, differentiation, and apoptosis (34). Recently, studies have shown that high expression of IL-1β in serum and tissues is associated with poor prognosis of patients. IL-1-mediated inflammation leads to the pathology of many diseases, especially MI (35). An article published in the *New England Journal of Medicine* has demonstrated that canakinumab, as a fully human monoclonal antibody targeting interleukin-1β, has been proven to lead to a significantly lower rate of the occurrence of MI (36). This evidence demonstrated that IL1B plays a key role in MI, which also supported the results of this study. In addition, TLR2 played a significant role in the process of atherosclerotic lesions and MI (37). Li et al. found that the increased expression of TLR2 supported the point that proinflammatory TLR was involved in the pathogenesis of MI. TLR2 was a predictive biomarker in elderly patients with MI (38). Consequently, IL1B and TLR2 were important biomarkers of MI.

From the results of immuno-infiltration analysis, multiple immune cell subtypes were discovered to be intimately involved in the critical biological processes of MI. A growth infiltration of activated mast cells, monocytes, and neutrophils and a reduced infiltration of resting mast cells, Tregs, and gamma delta T cells were detected to be potentially associated with the occurrence and development of MI. Furthermore, immune correlation analysis demonstrated that IL1B was positively correlated with activated mast cells, monocytes, and neutrophils and negatively correlated with resting mast cells, Tregs, and gamma delta T cells. Inflammatory and immune cells, including neutrophils, lymphocytes, and macrophages, have previously been proven to play an essential role in the development of cardiovascular disease (39, 40). In the early stages of MI, cardiomyocytes undergo necrosis, with severe sterile inflammation and a significant increase in monocytes and neutrophils in the blood. This also implies the initiation of intrinsic immunity (41). Infiltrated neutrophils in the infarct ozone can release reactive oxygen species and matrix-degrading enzymes, exacerbating myocardial damage (42). During the proliferative repair phase, CD4^+^ and CD8^+^ T cells, Tregs, and NK T cells can enter the infarcted area and promote the maturation and differentiation of these cells. The activation of Tregs is likely to be a promising treatment for MI to enhance the ability of cardiac repair and reduce MACE. In total, these types of infiltrating immune cells play an invaluable role in AMI and should be the focal point of future research.

The limitations of this study need to be recognized. To begin with, the design of this study was a retrospective cohort study. Thus, crucial clinical information could not be acquired. Finally, the function and the immune cell infiltration of IL1B and TLR2 in MI by bioinformatics analysis, SCS, and machine learning algorithms require the designation of prospective studies to validate our outcomes in the following days.

## Conclusion

This study identified two hub DEGs (i.e., IL1B and TLR2) and illustrated their potential roles in the diagnosis of MI to enhance our knowledge of the underlying molecular mechanism. Infiltrating immune cells played an important role in myocardial infarction. TCM, especially HF, was a potential drug for the treatment of MI.

## Data availability statement

The datasets presented in this study can be found in online repositories. The names of the repository/repositories and accession number(s) can be found in the article/Supplementary Material.

## Ethics statement

Ethical review and approval was not required for this study with human participants, in accordance with the local legislation and institutional requirements.

## Author contributions

QZ and DZ designed and wrote the original manuscript. QZ, YG, YP, and DW performed the experiments and wrote the original manuscript. DZ designed the experiments. DZ, QZ, BZ, and HL administered and coordinated the whole study project. All authors have read and agreed to the published version of the manuscript.

## Conflict of interest

The authors declare that the research was conducted in the absence of any commercial or financial relationships that could be construed as a potential conflict of interest.

## Publisher’s note

All claims expressed in this article are solely those of the authors and do not necessarily represent those of their affiliated organizations, or those of the publisher, the editors and the reviewers. Any product that may be evaluated in this article, or claim that may be made by its manufacturer, is not guaranteed or endorsed by the publisher.

## Footnotes

1
http://bis.zju.edu.cn/HCL/index.html

2
https://www.genecards.org/

3
https://metascape.org/gp/index.html#/main/step1

4
https://cn.string-db.org/

5
https://cibersortx.stanford.edu/

6
https://coremine.com/medical/#search

7
https://www.rcsb.org/
