# Supplementary figures and images for: Identification of hub biomarkers of myocardial infarction by single-cell sequencing, bioinformatics, and machine learning
Source: Front Cardiovasc Med. 2022 Jul 25;9:939972. doi: 10.3389/fcvm.2022.939972 (PMC9357907; doi:10.3389/fcvm.2022.939972)

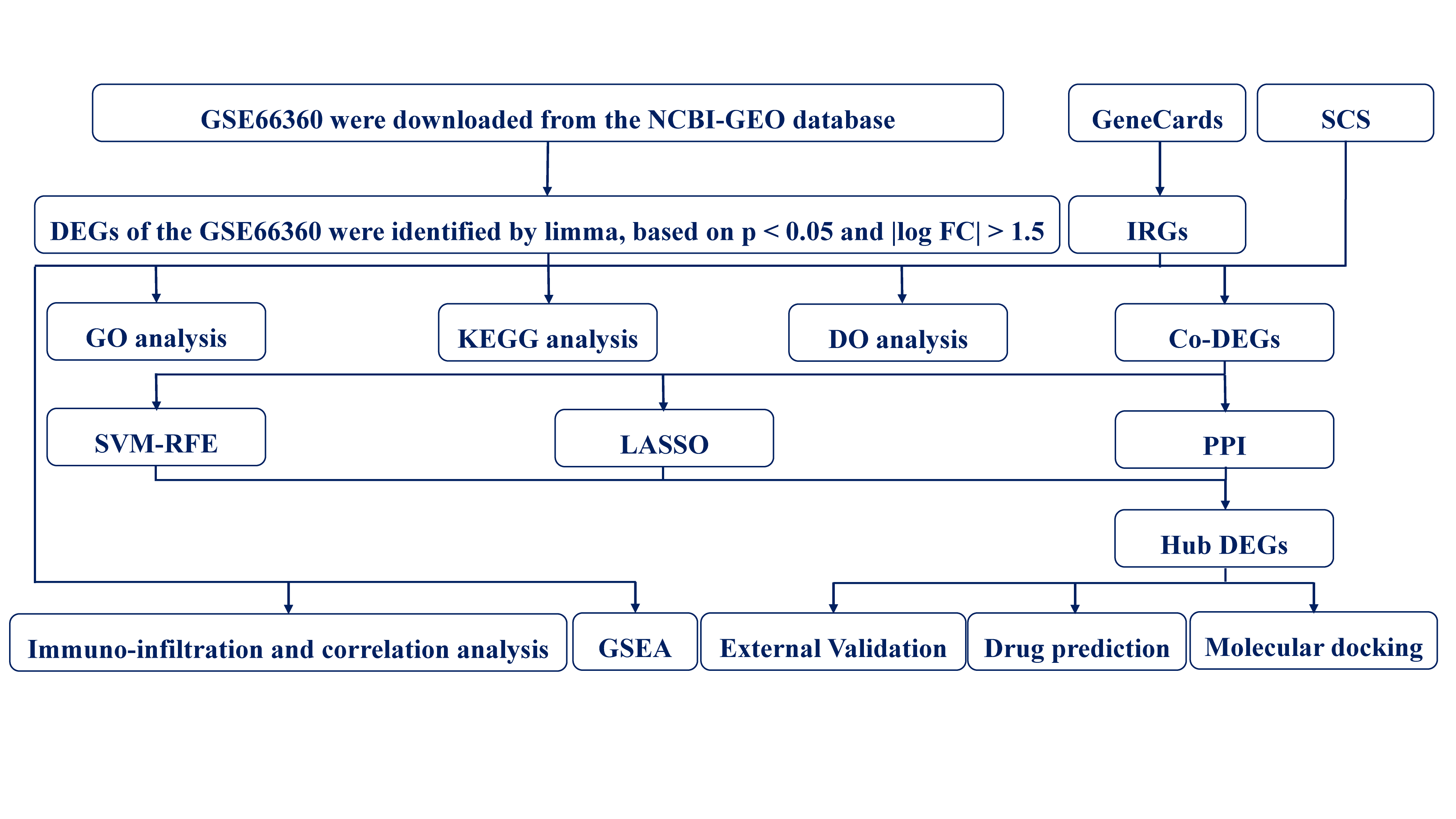

Supplement: Supplementary file 1 [file Data_Sheet_1.ZIP › Raw Data_FIGURE1_The flow chart of this study..tif]

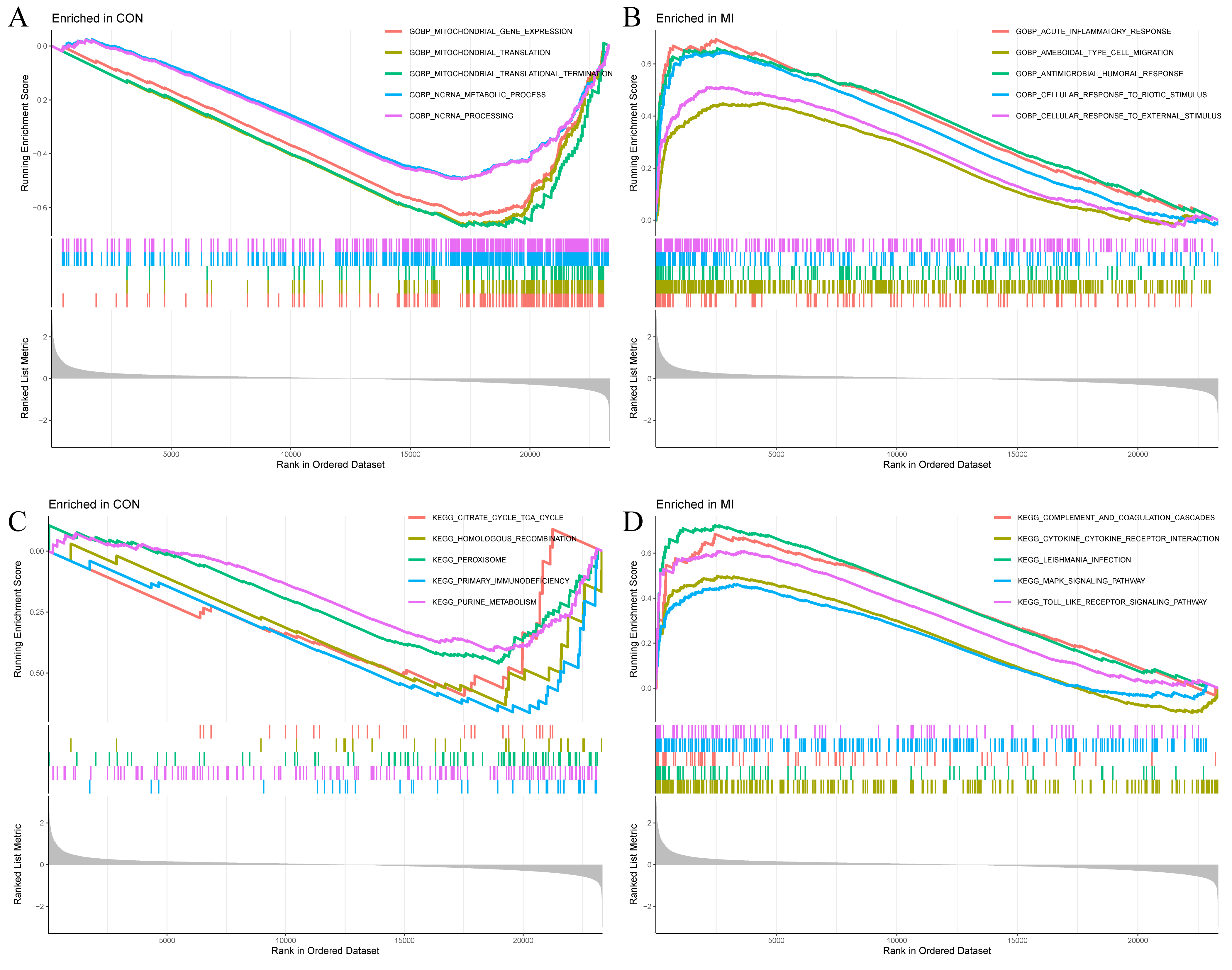

Supplement: Supplementary file 1 [file Data_Sheet_1.ZIP › Raw Data_FIGURE5_The Results of GSEA..tif]

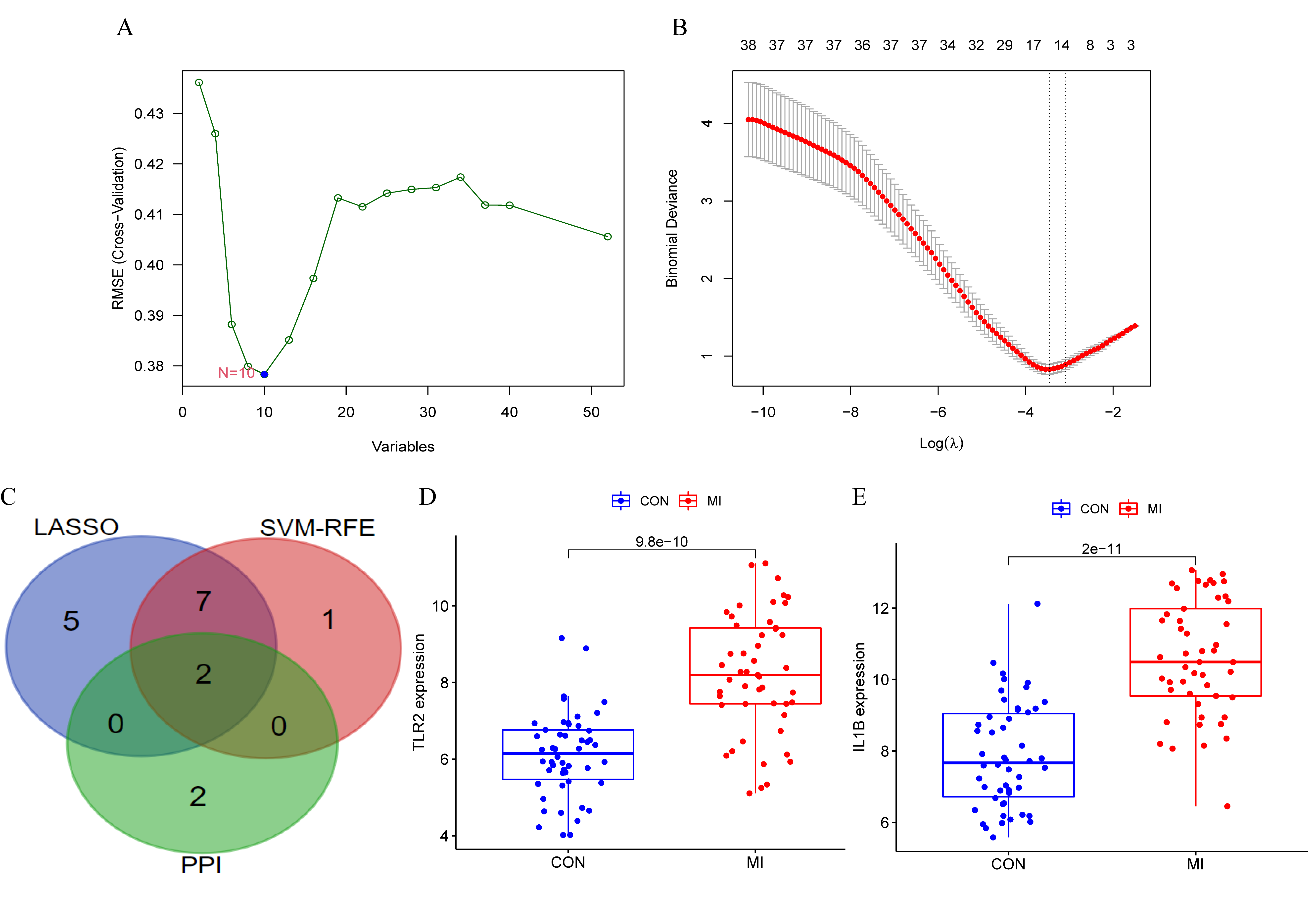

Supplement: Supplementary file 1 [file Data_Sheet_1.ZIP › Raw Data_FIGURE6_The screening of hub DEGs using machine learning and PPI..tif]

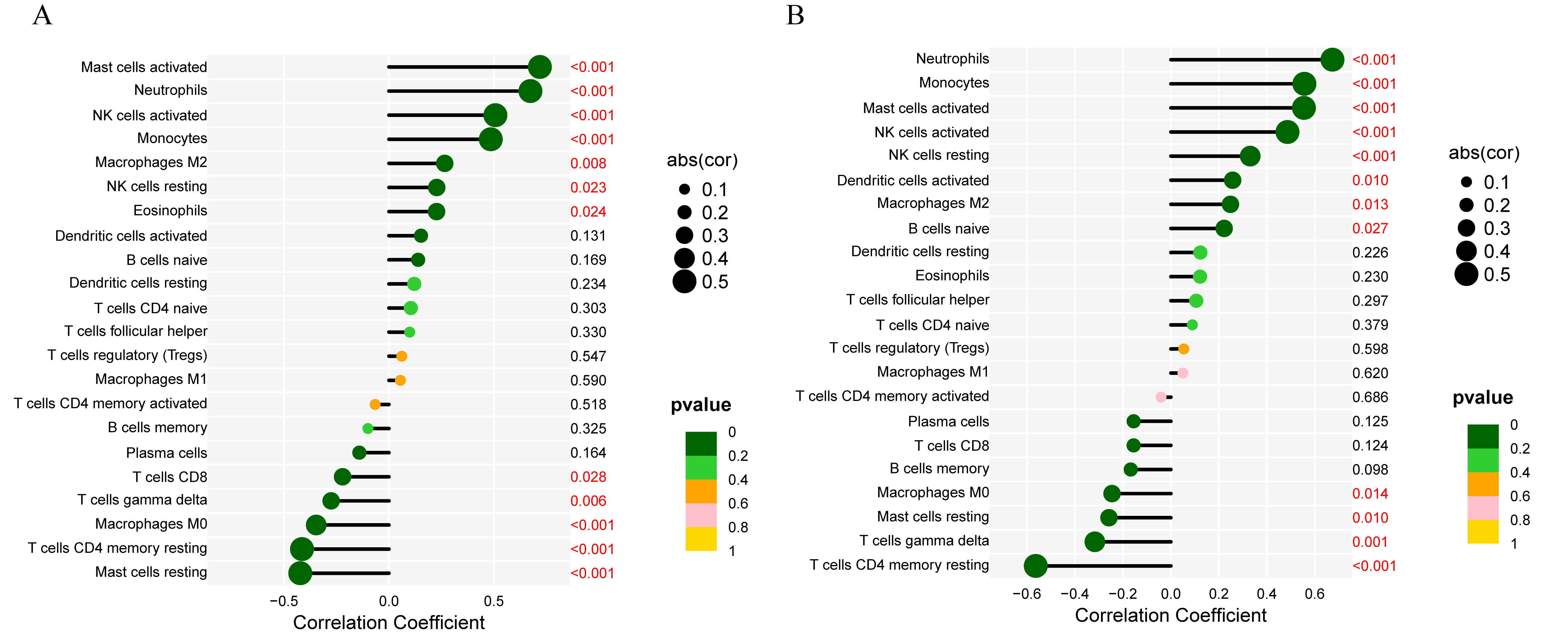

Supplement: Supplementary file 1 [file Data_Sheet_1.ZIP › Raw Data_FIGURE9_Correlation between IL1B, TLR2 and infiltrating immune cells..tif]
